# Endotoxin as a Marker for Water Quality

**DOI:** 10.3390/ijerph192416528

**Published:** 2022-12-09

**Authors:** Anas A. Sattar, Christian R. Good, Margaux Saletes, João Brandão, Simon K. Jackson

**Affiliations:** 1 Molendotech Limited, Brixham Laboratory, Blackball Lane, Freshwater Quarry, Brixham TQ5 8BA, UK; 2 Department of Environmental Health, National Institute of Health Dr. Ricardo, Avenida Padre Cruz, 1649-016 Lisboa, Portugal; 3 Centre for Environmental and Marine Studies (CESAM), Department of Animal Biology, University of Lisboa, 1749-016 Lisboa, Portugal; 4 School of Biomedical Science, University of Plymouth, Drake Circus, Plymouth PL4 8AA, UK

**Keywords:** BacterisK, endotoxin, faecal contamination, LAL assay, water testing, drinking water

## Abstract

Background: Water quality testing is vital to protect human health. Current testing relies mainly on culture-based detection of faecal indicator organisms such as *Escherichia coli (E.coli)*. However, bacterial cultures are a slow process, taking 24–48 h and requiring specialised laboratories and trained personnel. Access to such laboratories is often sparse in developing countries and there are many fatalities deriving from poor water quality. Endotoxin is a molecular component of Gram-negative bacterial cell walls and can be used to detect their presence in drinking water. Method: The current study used a novel assay (BacterisK) to rapidly detect endotoxin in various water samples and correlate the results with *E. coli* content measured by culture methods. The data generated by the BacterisK assay are presented as an ‘endotoxin risk’ (ER). Results: The ER values correlate with *E. coli* and thus endotoxin can be used as a marker of faecal contamination in water. Moreover, the BacterisK assay provides data in near real-time and can be used in situ allowing water quality testing at different spatial and temporal locations. Conclusion: We suggest that BacterisK can be used as a convenient risk assessment tool to assess water quality where results are required quickly or access to laboratories is lacking.

## 1. Introduction

Water is a vital part of human life, through drinking, washing, bathing to use in injectables. Water quality testing is thus a prerequisite for protecting human health. Unfortunately, many people worldwide do not have access to clean and safe drinking water, which results in waterborne bacterial infections with many fatalities associated [1].

At present, routine testing of water relies on the detection of indicator organisms as the only accepted means for assessing the sanitary quality of water supplies. Indicator organisms, such as *E. coli*, are used as a sign of faecal contamination of water rather than having to test for a myriad of specific pathogens. Yet, current culture-based methods for faecal organisms have several drawbacks as these indicators may reproduce in water and the culture results are retrospective, taking at least 24–48 h [2,3,4]. Moreover, although they indicate the presence of faecal contamination, other pathogens that are not necessarily of faecal nature, may go undetected [5]. In addition, the recent review of the ISO 9308 redefined its scope to cleaner waters (mostly with less than 100 colonies on chromogenic coliform agar) due to the background growth of less clean waters, which can interfere with the reliable enumeration of E. coli and coliform bacteria [6].

Although access to clean drinking water may be the norm in developed countries, in many developing countries people struggle to obtain access to safe water. According to the World Health Organisation (WHO) [7], 2.5 billion people have no access to improved sanitation and more than 1.5 million children die each year from diarrheal diseases. Additionally, the mortality of water-associated diseases exceeds 5 million people per year.

For Potable water, the WHO recommend a threshold of 0 CFU (Colony Forming Unit)/100 mL of *E. coli*, however, the WHO understand that achieving 0 CFU/100 mL *E. coli* may be challenging in many developing countries and rural areas. Therefore, the WHO assigned a health risk score for *E. coli* in drinking water: 0 CFU/100 mL (conformity), 1–10 CFU/100 mL (low risk), 10–100 CFU/100 mL (intermediate risk), 100–1000 CFU/100 mL(high risk), and >1000 CFU/100 mL (very high risk) [7,8].

A rapid and simple test of bacterial water quality should be a very useful tool, especially in disaster situations, such as floods or hurricanes, and in instances of treatment system failure. Several investigators [9,10,11,12,13] have suggested that the *Limulus amebocyte* lysate (LAL) assay for endotoxin may be a useful technique for rapidly determining bacterial biomass and quality of water. Endotoxin is the lipopolysaccharide (LPS) present in the outer membrane of Gram-negative bacteria and some cyanobacteria. While ingestion of large amounts of endotoxin (>1000 endotoxin units (EUs) can cause fever, diarrhoea, vomiting, acute respiratory illnesses, and lung inflammation, it can cause severe inflammatory reactions leading to shock, organ failure and death, if small amounts (<0.1 EU) enter sterile body cavities through inhalation or injection [3]. Endotoxins are therefore of immediate concern in many pharmaceutical industry water systems producing parenteral products because of these effects [3]. Endotoxin in pharmaceutical products for injection must be carefully monitored using the LAL assay which can detect picogram amounts of the molecule. Under these circumstances, the assay requires about 2 h to perform by skilled technicians in specialised laboratories.

Previous work by our group has shown the applicability of using endotoxin as a marker of faecal contamination of seawater [14] and that measuring endotoxin correlates with inflammatory effects of contaminated water samples [15]. The team at Molendotech have developed a near real-time assay (BacterisK) to detect endotoxin in water which can be conducted by non-specialist staff in situ. The advantage of measuring endotoxin as an indicator for contaminated water is that the test is specific for LPS, a compound which only naturally occurs in the cell walls of Gram-negative bacteria. LPS comprises a relatively constant proportion of a Gram-negative bacterial cell and Gram-negative bacteria account for 80 to 95% of the prokaryotes found in waters [16]. Moreover, endotoxin could indicate the presence of Gram-negative pathogens not detected by current culture of total coliforms or *E. coli*. Therefore, this novel assay would provide rapid testing in remote and disaster areas where access to laboratories and water testing facilities is challenging.

The present study was undertaken to test the applicability of the novel BacterisK assay as a rapid test of water quality and to correlate the amount of endotoxin in water with other measuring methods used in water quality assessment.

## 2. Materials and Methods

### 2.1. Water Sampling

A total of 69 environmental non-saline freshwater samples and potable tap water samples were collected from various locations in Southwest England (GPS coordinates are available as Supplementary Data). Multiple water samples (100 mL each) were taken from 10–30 cm below the surface of the water into sterile screw-capped pots. Tap water samples were collected by running the tap for 2–3 min and then collecting the sample in sterile screw-capped pots. Samples were immediately taken to the laboratory and processed within 6 h (minimum in duplicate) or kept at 4 °C and assayed overnight. Samples for LPS detection were aliquoted into endotoxin-free glass tubes (Lonza, Slough, UK) and preserved at 4 °C until assay in duplicate within 1 week [17].

### 2.2. Membrane Filtration Method

Water samples were filtered aseptically through a 0.45 μm membrane (Whatman, UK) and placed on Membrane Lauryl Sulphate agar for total coliforms and *E. coli* or Nutrient Agar for total aerobic bacterial count. Appropriate volumes (1, 10 and 100 mL) of the samples were aseptically filtered in duplicate using a vacuum pump attached to the water filtration system and results are expressed as CFU/100 mL. Standard Membrane Lauryl Sulphate total coliforms cultures were incubated aerobically at 35 °C for 24–48 h and for *E. coli* incubated aerobically at 35 °C for 4 h, then at 44 °C for 44 h while nutrient agar plates were incubated at 37 °C for 24–48 h. The numbers of colony-forming units (CFU) were calculated and numbers were expressed as CFU/100 mL [14].

### 2.3. BacterisK Assay

The BacterisK assay is based on an end-point endotoxin detection for use with environmental samples. The BacterisK assay, developed and provided by Molendotech Ltd., Brixham, UK, for the detection of endotoxin in environmental samples is based on the principle of the LAL assay. This is an enzyme cascade reaction triggered by endotoxin binding to Factor C in the LAL [18]. Subsequent enzymes in the LAL (factor B, Coagulogen) are activated and the latter cleaves the synthetic peptide-p-nitroanaline substrate resulting in the production of a purple colour after colour development.

The assay was performed following the kit instructions. Briefly, samples were diluted 1:100 by adding 10 μL of sample to 990 μL of Reagent W (pyrogen-free water) which is provided with the assay. Then, 100 μL of the diluted samples, a negative control (pyrogen-free water) and a positive control (Endotoxin standard from *E. coli* O55:B5) were transferred into fresh pyrogen-free glass tubes. Then, 100 μL of Reagent A and Reagent B were added to each tube and incubated at 37 °C for 16 min. Following the incubation, 500 μL of Reagent C, D and E were added consecutively. The completed reaction was transferred into semi-micro cuvettes and results were measured using a hand-held reader (Hach, Manchester, UK).

Results from the BacterisK assay are presented in ‘endotoxin risk’ (ER) units, which is an arbitrary scale derived from the colour change of the reagents in the end-point assay. Analysis of *E. coli* standard endotoxins of known concentration were also run at the same time, so that ER results could be linked to endotoxin concentration (Range 0–1.0 EU/mL~0–10,000 ER units). The assay protocol was designed to overcome effects of turbidity, pH, salinity etc. and is thus suitable for a broad range of water samples.

### 2.4. Kinetic-QCL™ LAL Assay

Selected water samples (*n* = 64) were also analysed using a kinetic LAL assay (kinetic-QCL™, Lonza, UK) according to the manufacturer’s instructions. This allowed a correlation of the endotoxin concentration (in EU/mL) with the ER values from the BacterisK assay.

### 2.5. Statistical Analysis

Statistical analysis was performed using Graphpad prism version 9. Results of selected water samples analysed using Kinetic-QCL™ were calculated using WinKQCL Endotoxin Detection and Analysis Software Version 5.3.3 (Lonza).

## 3. Results

**Figure 1:**
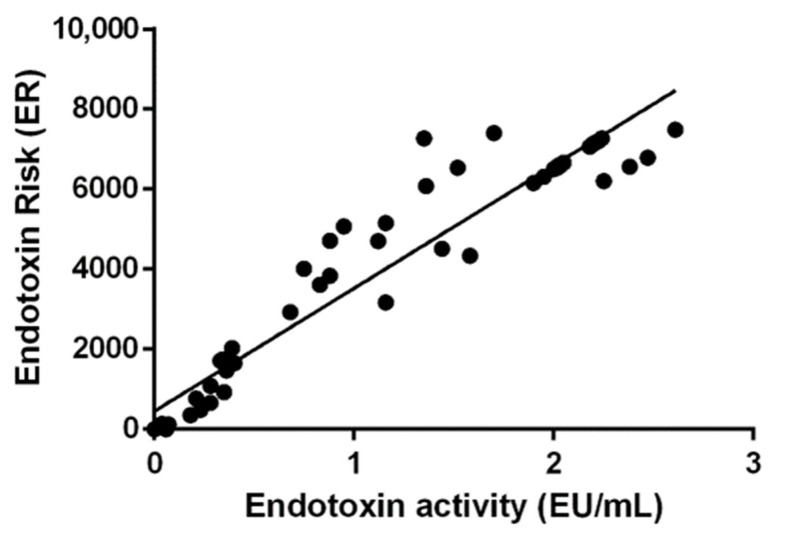
A Pearson correlation between the endotoxin units (EU/mL) and ER. Endotoxin unites were calculated using the Kinetic-QCL™ assay and compared to ER as calculated using BacterisK. *n* = 64, *p* < 0.0001, R^2^ = 0.929.

A total of 64 water samples were analysed by both the Kinetic-QCL™ assay and the BacterisK assay to establish a correlation between Endotoxin unites (EU/mL) and ER. The BacterisK assay directly correlates (*p* < 0.0001, R^2^ = 0.929) with endotoxin activity (Figure 1).

**Figure 2:**
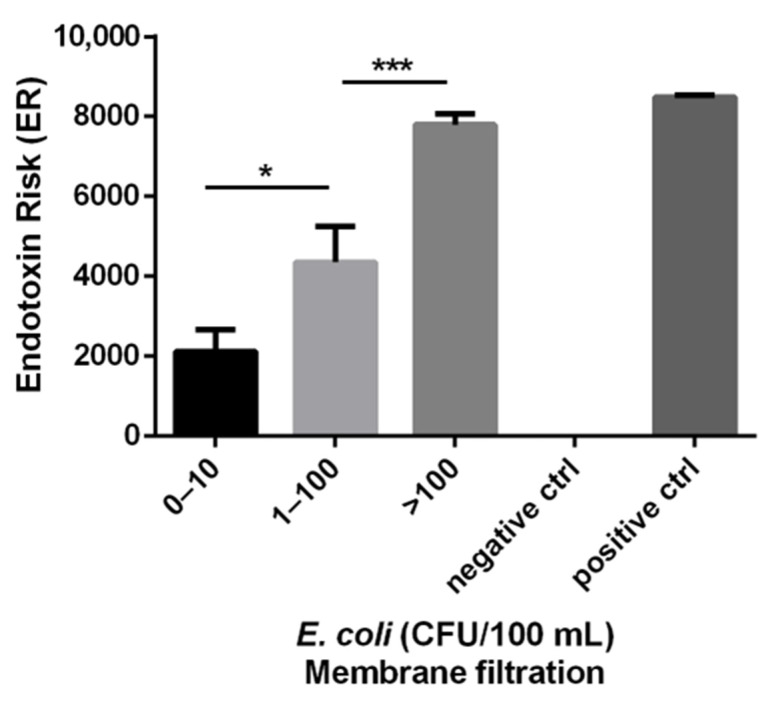
Relationship between *E. coli* (CFU/100 mL) and ER in various freshwater samples. *E. coli* CFU were enumerated using the standard Membrane filtration method. ER was obtained using the BacterisK assay. Each bar represents the mean ER + SEM for each group of *E. coli* CFU/100 mL. A total of 69 independent samples were analysed. The 0–10 group consisted of 23 samples, the 11–100 group consisted of 14 samples and the >100 group consisted of 32 samples. The negative control represents pyrogen-free water and the positive control represents purified endotoxin from *E. coli* O55:B5. * *p* < 0.05 and *** *p* < 0.001 as calculated using an unpaired *t*-test.

Water samples were classified into three groups based on the level of *E. coli,* 0–10, 11–100 and >100 CFU/100 mL according to the WHO recommendations [7,8], and the mean ER for each group are shown in Figure 2. The 0–10 CFU/100 mL group had a mean ER of 2118 with a standard error of the mean (SEM) of 550, the 11–100 CFU/100 mL group had a mean ER of 4356 with a SEM of 898 and the >100 CFU/100 mL group had a mean ER of 7796 with a SEM of 275. The 0–10 and 11–100 CFU/100 mL groups were significantly different (*p* < 0.05) as were the 11–100 and >100 CFU/100 mL groups (*p* < 0.001).

The BacterisK assay measures ER that can be used as a rapid determinant of water quality in terms of Gram-negative bacterial contamination. Using the data shown in Figure 2, ER ‘cut-off’ values that would allow water samples to be graded as ‘low risk’, ‘intermediate risk’ or ‘high risk’ with respect to probable bacterial contamination were determined. The ‘low risk’, ‘intermediate risk’ and ‘high risk’ groups equated to ER cut-off values of <500, 500–7000 and >7000, respectively. The lower cut-off value of <500 ER was determined to ensure no false negative results and is lower than the 2118 mean ER for the 0–10 CFU/100 mL group (Figure 2), while the upper cut-off value of >7000 ER was based on the 7796 mean ER for the >100 CFU/100 mL group (Figure 2). A Chi-squared analysis using these groupings provides a significant correlation (*p* < 0.0001) (Table 1).

From the *E. coli* culture analysis, 23 samples gave a count of less than 10 CFU/100 mL. Among them, 9 samples (39%) gave an ER result less than 500 ER on the BacterisK assay. The other 14 samples gave a result of >500 ER units. For the 11–100 CFU/100 mL category, composed of 14 samples, 9 of them had an ER result between 500 and 7000 and 5 others had an ER result above 7000. Importantly, no samples with *E. coli* culture of >10 CFU/100 mL gave a result less than 500 ER. Finally, in the >100 CFU/100 mL category, 100% of samples (32) gave a result of >500 ER units and 29 of these (90%) gave an ER result over 7000.

Fifteen samples were assessed in duplicate by the BacterisK assay to determine the repeatability. The mean and standard deviation were calculated to obtain a variation coefficient (Table 2). Global variation coefficient, obtained from the means of all variation coefficients (*n* = 15), is equal to 3%. As this result is less than 10%, the repeatability of the BacterisK assay is shown to be acceptable.

## 4. Discussion

According to the World Health Organization, more than 1.8 billion people use drinking-water sources that are contaminated with faeces [8]. Contaminated water is known to be linked to various diseases, such as cholera, diarrhoea, or dysentery. According to the WHO, the mortality of water-associated diseases exceeds 5 million people per year and 1.5 million children die annually from diarrhoea linked to unsafe drinking-water, sanitation, and hand hygiene [7]. Bacterial contamination of drinking water is a major contributor to water-borne diseases in rural areas of most developing countries where water sources are communally shared and exposed to multiple faecal-oral transmission pathways [7].

Since much of the microbiological water contamination is derived from human and animal faecal origin, monitoring water sources for faecal bacteria has become the practice to determine water quality. Detection of all possible pathogens in water would be too difficult, expensive and laborious; hence indicator organisms are used to infer the presence of faecal organisms which may include pathogens. The most used indicator bacteria is *E. coli*. which is usually only found associated with faecal matter from warm-blooded animals and humans [4,19,20].

Currently, the gold standard method to quantify these bacteria is the membrane filtration method. However, for shallow and surface waters the ISO 9308 is no longer applicable, due to a change in the growth medium described in the standard, from 2,3,5,-triphenyltetrazolium chloride (TTC) to Coliforms Chromogenic Agar (CCA). The latter is a more efficient medium, but this change rendered the ISO no longer applicable for use with recreational waters. This change caused some commotion on the enforcement of the current European Bathing Water directive because it was the reference method [7] and it must now be reviewed. Nonetheless, for in-scope waters, the main drawback of this technique is that results are still retrospective, taking between 24–48 h to have results. In addition, it requires sending samples to be processed in a laboratory with technically skilled staff.

Unfortunately, in low-income countries, especially in rural areas, access to a laboratory and expensive equipment is often an issue. Therefore, there is a pressing need for faster and more convenient methods to test water samples and determine a rapid risk analysis of water to be used for drinking. Ideally, such a rapid risk assessment test should be portable and be able to be used in field at the site of water sources as well as at distribution networks.

The patented BacterisK technology, developed by Molendotech, is a rapid, in situ testing kit that targets the presence of endotoxins as a biomarker for bacterial contamination. Therefore, BacterisK can provide a risk assessment for the presence of common pathogens in waterborne diseases, such as *E. coli*, but also all Gram-negative bacteria, including *Pseudomonas aeruginosa*, *Vibrio cholerae*, *Salmonella* and *Campylobacter*. The BacterisK technology is based on a colorimetric assay that takes less than thirty minutes to obtain results. BacterisK directly reports an ER value: the higher the value the higher the water contamination risk. However, it is important to show that such a rapid risk assessment tool has a correlation with faecal bacterial contamination. The present study was conducted to compare the ER with CFU/100 mL *E. coli* measured by the membrane filtration method.

Results from the current study show that the BacterisK assay can be used as a rapid risk assessment tool to indicate the level of faecal bacterial contamination of water.

Levels of ER correlated with the presence of *E. coli* over a large range from very low levels (0–10 CFU/100 mL) to high levels (>100 CFU/100 mL). Moreover, the BacterisK assay can be used to generate risk assessment in saline bathing water samples (Good et al., manuscript in preparation).

Currently, water quality control is monitored following the WHO guidelines [8]. Faecal indicator bacteria (FIB) are used as a proxy for the presence of faecal contamination in water resources. These indicators include *Escherichia coli*, total coliforms, faecal coliforms and *Enterococci*. Regarding the presence of *E. coli*, WHO guidelines recommend a threshold of 0 CFU (Colony Forming Unit)/100 mL, However, the WHO understand that 0 CFU/100 mL *E. coli* may not be easily achievable in many developing countries and has assigned a health risk score for *E. coli* in drinking water: 0 CFU/100 mL (conformity), 1–10 CFU/100 mL (low risk), 10–100 CFU/100 mL (intermediate risk), >100 CFU/100 mL (high risk) [7,8]. Results using the BacterisK assay show it to be useful in predicting risk bands aligned with these WHO guidelines. Our results indicate potential thresholds or cut-offs for the risk evaluation of water <500 ER for low risk, 500–7000 for medium risk, and >7000 high risk (Figure 2, Table 1). While the ER values correlated well with *E. coli* CFU, it should be remembered that these assays measure different targets (live bacteria vs. molecular endotoxin) and therefore we would not expect a strong correlation between their values. However, the results do indicate a good association and the predictive value of the ER assessment would suggest this as a useful measure of water quality.

It can be seen from Figure 2 and Table 1 that some samples produced a positive ER response above the 500 threshold yet their *E. coli* content was low (<10 CFU/100 mL). In essence, these would be classified as ‘false positives’. This is expected due to the BacterisK assay detecting a molecule, endotoxin, present in all Gram-negative bacteria and would indicate a potential risk from these samples due to contamination with other species. Investigation of these samples indeed showed the presence of other coliform species (results not shown). False-positives (indicators present in the absence of detectable pathogens) also occur fairly often with *E. coli* measurement. Although they cause false alarms, these false positives are tolerated. Public health officials are willing to accept reasonable numbers of false-positives because they err on the side of public health. These false positives alone will cause correlation coefficients to be low. Importantly, in the current study, none of the samples had an ER value below the 500 ER cut off and an *E. coli* result of >10 CFU/100 mL. This suggests that the BacterisK method does not produce false negative results, which is more important for public health assessment. With a threshold of 500 ER and 10 CFU/100 mL *E. coli*, the BacterisK assay has a true positive predictive value of 100%.

In general, coliform indicators alone cannot provide conclusive, non-site-specific and non-pathogen-specific information about the presence and/or concentrations of most important pathogens in surface waters suitable for irrigation [19,20]. Nonetheless, *E. coli* concentrations are currently used by regulatory agencies to assess the presence of faecal contamination and other pathogens in freshwaters. These organisms are monitored primarily as an index of suspicion: if faecal contamination is present, pathogens may also be present. It should be noted that BacterisK will respond to endotoxin from all Gram-negative organisms present in the sample and might therefore give a risk assessment from non-faecal organisms. The presence of human pathogens that are not of faecal origin represent a significant concern in many areas. Pathogenic groups of Vibrio bacteria, such as *V. vulnificus* and *V. parahaemolyticus*, for example, are naturally occurring and cause numerous cases of gastroenteritis and wound infections [21].

The results presented in this study highlight the advantage of the BacterisK assay as a rapid risk assessment of water contamination with faecal and other Gram-negative bacteria. The results obtained in 30 min provide a near real-time monitoring of the water contamination as compared with 24–48 h for the culture of *E. coli* as is the usual practice. In addition to the rapid results, the portability of the BacterisK assay mean it can be performed in situ at the water being tested. The assay is relatively simple to perform and does not require sterility needed for the membrane filtration method. The rapid results with low COV in repeatability coupled with the portability allow the BacterisK assay to be used for source monitoring programs. The BacterisK assay is also in line with the United Nations vision of ensuring availability and sustainable management of water and sanitation for all (SDG 6); where rapid testing could provide valuable information to the users and potentially reduce health risks.

Current drinking water compliance monitoring using indicator organisms may not provide effective protection of public health and waterborne outbreaks remain common, even in high-income countries [22]. Delays resulting from culture methods also limit the ability to rapidly communicate the risks to local communities. To address some of these limitations with FIB monitoring, the WHO recommends a risk-based management approach to ensure water safety [7].

Other rapid methods that have been used for water quality include fluorescence and ATP measurement. Intrinsic fluorescence from organic matter at excitation 280 nm and emission at 350 nm due to tryptophan like fluorescence (TLF) can be correlated with biochemical oxygen demand. Studies have suggested that TLF can be correlated with microbial contaminants and *E. coli*. However, the number of compounds that can potentially give fluorescence in this region and the number of potential interferences to fluorescence limit the correlation with *E. coli* numbers [23]. Moreover, metals may quench the fluorescence while high dissolved organic carbon (DOC) and nitrate can give high TLF that do not correlate with high fecal indicator organisms [24,25]. The complexity and limitations coupled with the high cost of the instruments, particularly for online measurements, may limit their use in microbial water testing [26].

ATP bioluminescence can measure cellular material through measuring ATP levels expressed in relative light units. However, the correlation between ATP and the presence of microorganisms in water systems was found not to be significant [26]. Limitations to the ATP method include that it does not easily distinguish ATP from microorganisms, animals, and plants while luminescence from other sources can affect the actual ATP bioluminescence readings [26].

## 5. Conclusions

In conclusion, the present study was undertaken to assess the suitability of the novel BacterisK assay as a rapid test of water quality and to correlate the level of endotoxin in water with *E. coli*. This study has shown the applicability of rapid, on-site monitoring of bacterial endotoxin to give a near real-time assessment of water quality risk. In addition to being a useful tool for rapid fecal water contamination assessment and source tracking in a wide variety of settings, the technology is particularly useful for screening water contamination in low-resource countries and disaster relief situations that require a quick and simple assessment method for rapid and continuous screening of surface and groundwater quality [7,27]. The rapid assessment of bacterial endotoxin by BacterisK to provide a correlate of fecal indicator organisms such as *E. coli* has several advantages including portability, ease of use by non-technical experts, simple training requirements and the production of immediate results.

## Figures and Tables

**Table 1:** Chi-squared analysis of *E. coli* (CFU/100 mL) vs. ER cut-off values from the freshwater samples presented in Figure 1. Χ^2^ = 47.57, Degrees of freedom = 4, *p*-value = 0.0001.

	*E. coli* CFU/100 mL of Water			
	0–10 (Low Risk)	11–100 (Intermediate Risk)	>100 (High Risk)	Total
**<500 ER**	9	0	0	9
**500–7000 ER**	12	9	3	24
**>7000 ER**	2	5	29	36
**Total**	23	14	32	69

**Table 2:** Repeatability data. Samples were assessed in duplicate by the BacterisK assay to determine the repeatability. Global variation coefficient, obtained from the means of all variation coefficients (*n* = 15), is equal to 3%.

Sample ID	First Result	Second Result	Mean	Standard Deviation	Variation Coefficient
31	1770	2085	1928	223	12%
32	1080	1120	1100	28	3%
33	2650	2470	2560	127	5%
34	6155	7075	6615	651	10%
35	4840	4830	4835	7	0%
36	3930	4610	4270	481	11%
37	8485	8505	8495	14	0%
38	7365	7530	7448	117	2%
39	7755	7795	7775	28	0%
40	8320	8320	8320	0	0%
41	8280	8385	8333	74	1%
42	8495	8495	8495	0	0%
44	8395	8365	8380	21	0%
45	8445	8455	8450	7	0%
47	8415	8415	8415	0	0%
					**Mean: 3%**

## Data Availability

Data is stored on Molendotech′s private data repository and can be provided upon request.

## Supplementary Materials

The following supporting information can be downloaded at: https://www.mdpi.com/article/10.3390/ijerph192416528/s1.
