# Supplementary material for: Endotoxin as a Marker for Water Quality
Source: Int J Environ Res Public Health. 2022 Dec 9;19(24):16528. doi: 10.3390/ijerph192416528 (PMC9778876; doi:10.3390/ijerph192416528)
Supplement: Supplementary file 1 [file ijerph-19-16528-s001.zip › ijerph-2059944-supplementary.pdf]

Sample ID	GPS coordinates	Water source
1	50.589957717801084, -3.7317005371663745	Spring
2	50.59512067611063, -3.764340943457731	River
3	50.58219744125952, -3.7925480118514585	Spring
4	50.59489031407215, -3.7925480120742154	Spring
5	50.368302926894366, -3.5330117472592155	Spring
6	50.36780415210376, -3.5259896385522955	Spring
7	50.368511550787964, -3.5329015926405987	Pond
8	50.36780415210376, -3.5259896385522955	Spring
9	50.40148030211824, -3.517606329686128	Tap water
10	50.40495490592173, -3.554036225789188	Stream
11	51.503165061444825, -2.585980959468567	Tap water
12	50.36780415210376, -3.5259896385522955	Spring
13	50.368511550787964, -3.5329015926405987	Pond
14	50.368302926894366, -3.5330117472592155	Spring
15	50.36780415210376, -3.5259896385522955	Spring
16	50.64211409537967, -3.6885416255295382	Lake/reservoir
17	50.62825825126938, -3.690211423821171	Lake/reservoir
18	50.53633630722263, -3.649857748491364	Tap water
19	50.63158891807355, -3.683138301837764	Lake/reservoir
20	50.43939488023677, -3.564764674493676	Tap water
21	51.42420739919195, -2.857632538115001	River
22	51.503165061444825, -2.585980959468567	Tap water
23	51.503165061444825, -2.585980959468567	Tap water
24	51.48980440269506, -2.5508544724232123	Pond
25	50.42777947408942, -3.6817516129618757	River
26	50.42221556990036, -3.568919343781126	Pond
27	50.39115819468948, -4.156867582947575	Tap water
28	50.43939488023677, -3.564764674493676	Tap water
29	50.352206209460796, -3.565925887771069	Stream
30	50.387691944696854, -3.5187395325748914	River
31	52.18604193752356, 0.16719376808552333	Pond
32	52.18964748241256, 0.1685464060099242	Stream
33	52.19206717625702, 0.16571804294058828	Pond
34	52.18582682047728, 0.17005812028878253	Stream
35	52.19603392740351, 0.1167938043470293	River
36	51.503165061444825, -2.585980959468567	Tap water
37	51.230240889080044, -2.307793448037633	River
38	51.23180287877024, -2.304645077067615	Stream
39	50.56199436505669, -3.743991488557981	Spring
40	50.60693226716691, -3.7567450111353633	Spring

41	50.60109671522794, -3.735046271649488	Spring
42	50.60828615851925, -3.7285440947874307	Spring
43	50.60966788930623, -3.726211251949554	Spring
44	50.606863570735925, -3.725402342277685	River
45	50.61062329504194, -3.7265940161637983	River
46	50.55140217613052, -3.772861373516643	Stream
47	50.54316196339451, -3.751326858312035	Stream
48	50.40268759062639, -4.0869497657376135	River
49	50.408026759856675, -4.085358582365627	River
50	50.41011453951882, -4.077870660615104	River
51	50.41378284381124, -4.077355865994755	River
52	50.40820571600364, -4.0795086434980306	River
53	50.525352642978135, -3.626377386643733	Stream
54	50.5249747991845, -3.6282450960404704	River
55	50.526613495128814, -3.644498108467573	River
56	50.40268759062639, -4.0869497657376135	River
57	50.408026759856675, -4.085358582365627	River
58	50.41011453951882, -4.077870660615104	River
59	50.41378284381124, -4.077355865994755	River
60	50.40820571600364, -4.0795086434980306	River
61	50.52458570553273, -3.638124416342096	Stream
62	50.525352642978135, -3.626377386643733	Stream
63	50.527131377379426, -3.6333526930625535	River
64	50.526613495128814, -3.644498108467573	River
65	50.5249747991845, -3.6282450960404704	River
66	50.39115819468948, -4.156867582947575	Tap water
67	50.53633630722263, -3.649857748491364	Tap water
68	50.40148030211824, -3.517606329686128	Tap water
69	50.40148030211824, -3.517606329686128	Tap water
